# Cannabinoid-Induced Conditioned Place Preference, Intravenous Self-Administration, and Behavioral Stimulation Influenced by Ghrelin Receptor Antagonism in Rats

**DOI:** 10.3390/ijms22052397

**Published:** 2021-02-27

**Authors:** Chrysostomos Charalambous, Tereza Havlickova, Marek Lapka, Nina Puskina, Romana Šlamberová, Martin Kuchar, Magdalena Sustkova-Fiserova

**Affiliations:** 1 Department of Pharmacology, Third Faculty of Medicine, Charles University, Ruska 87, 100 00 Prague 10, Czech Republic; chrysostomos.charalambous@lf3.cuni.cz (C.C.); terez.hav@gmail.com (T.H.); marek.lapka@centrum.cz (M.L.); 2 Department of Addictology, First Faculty of Medicine, Charles University, Apolinarska 4, 128 00 Prague 2, Czech Republic; nina.puskina@seznam.cz; 3 Department of Physiology, Third Faculty of Medicine, Charles University, Ke Karlovu 4, 120 00 Prague 2, Czech Republic; romana.slamberova@lf3.cuni.cz; 4 Forensic Laboratory of Biologically Active Substances, Department of Chemistry of Natural Compounds, University of Chemistry and Technology Prague, Technicka 5, 166 28 Prague 6, Czech Republic; martin.kuchar@vscht.cz

**Keywords:** tetrahydrocannabinol (THC), synthetic cannabinoid, WIN55,212-2, ghrelin antagonism, addiction, intravenous self-administration, conditioned place preference, behavioral stimulation

## Abstract

Cannabis/cannabinoids are widely used for recreational and therapy purposes, but their risks are largely disregarded. However, cannabinoid-associated use disorders and dependence are alarmingly increasing and an effective treatment is lacking. Recently, the growth hormone secretagogue receptor (GHSR1A) antagonism was proposed as a promising mechanism for drug addiction therapy. However, the role of GHS-R1A and its endogenous ligand ghrelin in cannabinoid abuse remains unclear. Therefore, the aim of our study was to investigate whether the GHS-R1A antagonist JMV2959 could reduce the tetrahydrocannabinol (THC)-induced conditioned place preference (CPP) and behavioral stimulation, the WIN55,212-2 intravenous self-administration (IVSA), and the tendency to relapse. Following an ongoing WIN55,212-2 self-administration, JMV2959 3 mg/kg was administered intraperitoneally 20 min before three consequent daily 120-min IVSA sessions under a fixed ratio FR1, which significantly reduced the number of the active lever-pressing, the number of infusions, and the cannabinoid intake. Pretreatment with JMV2959 suggested reduction of the WIN55,212-2-seeking/relapse-like behavior tested in rats on the twelfth day of the forced abstinence period. On the contrary, pretreatment with ghrelin significantly increased the cannabinoid IVSA as well as enhanced the relapse-like behavior. Co-administration of ghrelin with JMV2959 abolished/reduced the significant efficacy of the GHS-R1A antagonist in the cannabinoid IVSA. Pretreatment with JMV2959 significantly and dose-dependently reduced the manifestation of THC-induced CPP. The THC-CPP development was reduced after the simultaneous administration of JMV2959 with THC during conditioning. JMV2959 also significantly reduced the THC-induced behavioral stimulation in the LABORAS cage. Our findings suggest that GHS-R1A importantly participates in the rewarding/reinforcing effects of cannabinoids.

## 1. Introduction

In the long term, cannabinoids have been the most widely used illegal drugs in Europe. Abused cannabinoids beside the natural constituents of *Cannabis sativa*/cannabis also include a number of synthetic cannabinoids used in different ways, e.g., as “spice” in herbal mixtures, infused papers, or even as adulterating cannabis with synthetic cannabinoids. From 2002 to 2019, more than 180 synthetic cannabinoids of various chemical structures, including aminoalkylindoles, were detected on the drug market by the European Monitoring Centre for Drugs and Drug Addiction (EMCDDA) [1]. Social, medical, and legal acceptance of cannabis has grown dramatically during the past 15 years in Europe and North America. The medical and recreational use of cannabis has also increased, but the public proportion that perceives important harms from cannabis/cannabinoids use has decreased [1,2]. In Europe, including the Czech Republic, a prevailing supply of high-potent/tetrahydrocannabinol (THC) strains of cannabis has existed over the last few years, linked with increased risks of cannabis use disorder, which includes uncontrolled drug-seeking and withdrawal symptoms, psychotic disorders, dysphoria, sleep and eating disorders, etc. [1,3,4]. Recently, it has been estimated that about 9% of chronic cannabis users display characteristic signs and symptoms of dependence according to the Diagnostic and Statistical Manual of Mental Health of the World Health Organization (WHO/DSM-IV) criteria [1,4]. Similar potential risks have also been associated with many new synthetic cannabinoids, including aminoalkylindole derivatives, which have been broadly abused in Europe and elsewhere [1]. Currently, no specific pharmacotherapies have been approved for cannabinoid/cannabis use disorder and dependence; thus, cannabinoid addiction treatment remains exclusively symptomatic, unsatisfactory, and with a low relapse prevention [5]. Therefore, new effective treatment strategies are constantly being researched.

Recently, the growth hormone secretagogue receptor (GHS-R1A) antagonism was suggested to be a promising mechanism for drug addiction treatment (firstly suggested by Jerlhag et al. 2006) [6]. The endogenous ligand of GHS-R1A, an acylated ghrelin, is known as a peptide modulator of systemic metabolism, food intake, and further various peripheral and central physiological functions, such as inflammation, thermogenesis, cardiac output and contractility, depression, sleep–wake rhythm, neuroprotection, memory, reward behavior, etc. [7,8,9]. An increasing number of animal studies has described the important role of ghrelin/GHS-R1A in alcohol, psychostimulants, and in opioid reward/reinforcement effects (see reviews) [10,11,12,13]. One GHS-R1A-inverse agonist (PF5190457) is being tested in initial clinical studies involving alcohol-dependent patients [14]. However, the available literature implicating ghrelin in cannabis/cannabinoid use disorders is currently limited and inconclusive [14]. It was described that a single intra-gastric administration of alcohol extract of *Cannabis sativa* [15], as well as a single intraperitoneal injection of synthetic cannabinoid CB1 receptor agonists, methanandamide, and CP55,940 [16], had increased total ghrelin blood concentrations and food intake in rats. Increased blood levels of total ghrelin were also observed after oral administration of cannabis in healthy adult cannabis users [17] and in chronic THC smoking HIV-infected men [18].

Cannabis/cannabinoids mediate their effects by stimulating the endocannabinoid system, characterized by great complexity, central and peripheral effects, several binding sites, and series of endogenous ligands/endocannabinoids [4,19,20]. Beside other roles, the endocannabinoid system importantly participates in metabolism, peripheral and central homeostatic as well as non-homeostatic/hedonic food intake, and drug abuse, similarly to the ghrelin system. Particularly, cannabinoid CB1R and ghrelin/GHS-R1A receptors are distributed within overlapping brain regions crucial for feeding (hypothalamus) and reward/reinforcement (ventral tegmental area/VTA, nucleus accumbens/NAC) [4,20,21]. Based on the current available animal studies, it appears that the endogenous cannabinoid system and the ghrelin system importantly mutually interact in the regulation of food intake on a base of homeostatic as well as hedonic principles [22,23,24].

Lately, it has been described that the pharmacological blockade of the CB1R via rimonabant administration attenuated the ghrelin-induced activation of the mesolimbic dopamine system in mice [25]. In our previous microdialysis studies, we used the GHS-R1A antagonist, a non-peptidic triazole derivate JMV2959 [26,27], in opioid addiction models, and our findings suggested that there are significant interactions among ghrelin/GHS-R1A and endocannabinoids (arachidonoyl ethanolamide/anandamide/AEA and 2-arachidonoylglycerol/2-AG) besides dopamine, opioid, and γ-aminobutyric acid/GABA systems within the rat mesolimbic structures (VTA and NAC) [28,29,30,31,32]. Recently, we also documented that JMV2959 (3 mg/kg i.p.) pretreatment significantly decreased the WIN55,212-2-induced accumbens dopamine efflux (WIN55,212-2 administered into the posterior VTA) (published in this Special Issue [33]). Thus, it may be presumed that ghrelin/GHS-R1A also plays an important role in cannabinoid/cannabis reinforcement pro-addictive effects; however, a clear evidence supporting this in more specific addiction models is currently missing.

Therefore, the aim of our present study was to test if the GHS-R1A antagonist would reduce the cannabinoid-induced conditioned place preference (CPP), the behavioral stimulation, the intravenous self-administration (IVSA), and the relapse-like/drug-seeking behavior in rats. In accordance with the literature, we have chosen reliable experimental cannabinoid models, which would produce cannabinoid/CB1R agonists reinforcing pro-addictive effects, which could be antagonized. Tetrahydrocannabinol (THC), the main psychotropic constituent of cannabis, was chosen for our CPP and behavioral experiments and a synthetic aminoalkylindole cannabinoid WIN55,212-2 [34,35] for the IVSA experiment, because in the contradistinction to THC, WIN55,212-2 had been reliably self-administered in rodents/rats [36,37,38]. Both cannabinoids, classical THC, and aminoalkylindole WIN55,212-2 differ distinctly in structure. WIN55,212-2 is less lipophilic, is eliminated more readily than THC [39,40], and precipitates more noticeable spontaneous withdrawal symptoms in rats [40,41]; however, both cannabinoids elicit comparable effectiveness in tests of hypothermia, analgesia, catalepsy, and locomotor suppression [42]. The THC is considered as a partial CB1R agonist [4,43], while WIN55.212-2 acts with higher affinity on the CB1R as a full agonist [35]. Thus, the WIN55,212-2 IVSA rat model is suitable for testing the GHS-R1A involvement in the general cannabinoid/CB1R agonist reinforcing pro-addictive effects, but the transferability of the obtained results to cannabis (marihuana, hashish) use has certain limits.

## 2. Results

### 2.1. JMV2959 Effects on Manifestation and Development of THC-Induced Conditioned Place Preference (CPP)

**Figure 1:**
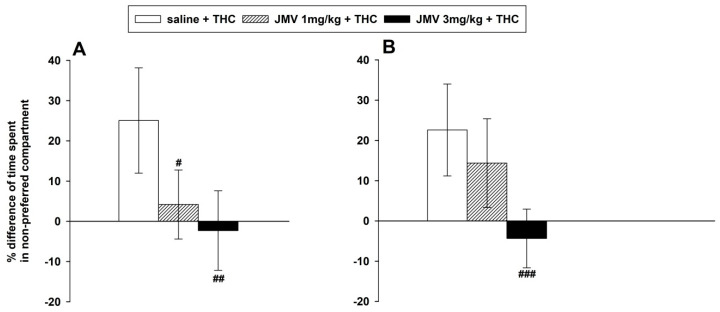
Effects of JMV2959 on tetrahydrocannabinol (THC)-induced conditioned place preference (CPP) in rats. In graph (**A**), JMV2959 (0, 1, 3 mg/kg i.p.) was administered in a single dose 20 min before the final testing after 8 days of conditioning with THC (0.3 mg/kg i.p.) (saline *n* = 11; JMV2959 groups *n* = 8; means ± SEM). In graph (**B**), JMV2959 (0, 1, 3 mg/kg i.p.) was administered repeatedly during the 8 days conditioning together with THC (0.3 mg/kg i.p.) (saline *n* = 10; JMV2959 groups *n* = 9; means ± SEM). The results are presented as follows: Saline + THC (open bar), JMV2959 1 mg/kg + THC (striped bar), JMV2959 3 mg/kg + THC (filled bar). CPP was calculated as the difference in percentage of total (1200 s) time spent in the THC-paired (i.e., least preferred) compartment during the post-conditioning and/minus the pre-conditioning session. The effects of JMV2959 pretreatments in comparison to the saline group are expressed as # *p* < 0.05, ## *p* < 0.01, ### *p* < 0.001. The results are presented as group means with 95% confidence intervals.

The CPP was calculated as the difference in the percentage of total (1200 s) time spent in the THC-paired (i.e., least preferred) compartment during the post-conditioning session (day 10) and/minus the pre-conditioning session (day 1); eight days of THC-conditioning were used. The established THC-induced CPP manifestation was significantly and dose-dependently attenuated by 1 and 3 mg/kg JMV2959 when administered 20 min before testing on the post-conditioning day: F2,24 = 8.65, *p* < 0.001 (see Figure 1A). When the higher dose 3 mg/kg JMV2959 was repeatedly administered together with THC during conditioning, the development of THC-CPP was significantly reduced: F2,25 = 9.52, *p* < 0.001; the effect of the lower dose (1 mg/kg) was not significant (see Figure 1B). The JMV2959 doses (1 and 3 mg/kg i.p.) did not significantly influence the rat locomotor behavior within the tested period in our previous study [28]. JMV2959 alone did not induce any CPP [44]; therefore, this experiment was not included.

In the Supplementary Information, the results of both CPP experiments were processed differently but with comparable findings; the data illustrated in Figure S1 are expressed as absolute values of time spent in the non-preferred/THC-paired compartment before and after conditioning. 

### 2.2. JMV2959 Effects on THC-Induced Behavioral Stimulation (LABORAS Cage)

**Figure 2:**
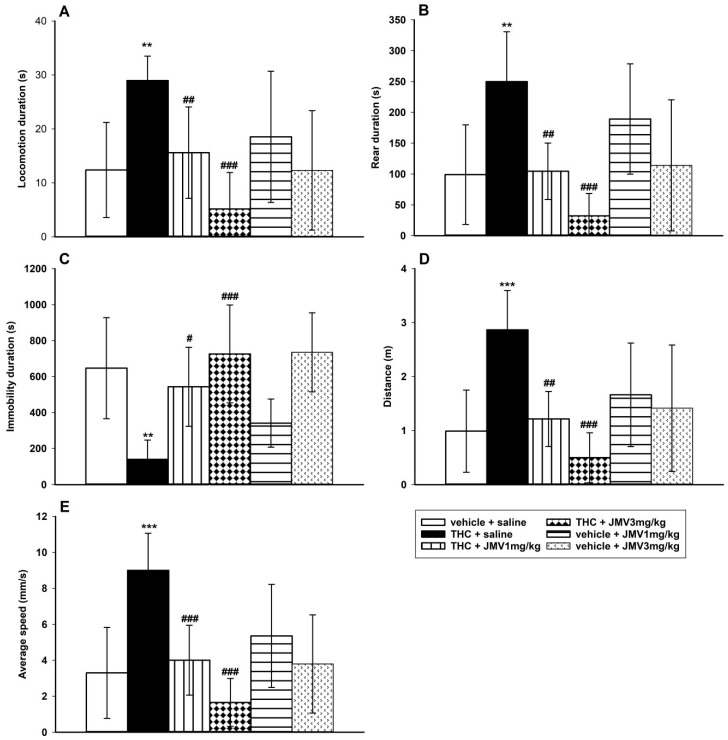
Effects of JMV2959 on the THC-induced behavioral changes in rats in the LABORAS cage. JMV2959 (0, 1, 3 mg/kg i.p.) was administered immediately before placing the rats into the cage and after 20 min of habituation, a stimulatory THC dose 0.1 mg/kg or saline was administered intraperitoneally. After another 20 min of habituation, behavior monitoring started and lasted for 20 min (20–40 min after THC administration). Changes in locomotion duration (**A**), rear duration (**B**), immobility duration (**C**), distance traveled (**D**), and average speed overall (**E**) are illustrated as follows: saline + vehicle (open bar) (*n* = 9), saline + THC (filled bar) (*n* = 7),1 mg/kg + THC (vertically striped bar) (*n* = 8), JMV2959 3 mg/kg + THC (diamond bar) (*n* = 8), JMV2959 1mg/kg + vehicle (horizontally striped bar) (*n* = 4), JMV2959 3 mg/kg + vehicle (little arrows bar) (*n* = 8). The JMV2959 pretreatment effects in comparison to saline + THC group are expressed as # *p* < 0.05, ## *p* < 0.01, ### *p* < 0.001. Differences between groups in comparison to vehicle + saline group are expressed as ** *p* < 0.01, *** *p* < 0.001. The results are presented as group means with 95% confidence intervals.

In comparison to the vehicle + saline group, the 0.1 mg/kg i.p. THC dose produced significant behavioral stimulation in the habituated rats monitored using the LABORAS cage within 20–40 min after the THC administration (See Figure 2), which is in accordance with the literature [45,46]. Following THC administration, the locomotion, rear, distance traveled, and average speed were significantly increased, while immobility was significantly decreased in comparison to the saline + vehicle group. The 1 or 3 mg/kg JMV2959 administered 20 min before THC, significantly and dose-dependently reduced the THC-induced changes in all monitored parameters using one-way ANOVA followed by Holm–Sidak test, specifically: locomotion duration (F5,38 = 4.66, *p* = 0.002) (see Figure 2A), rear duration (F5,38 = 5.37, *p* < 0.001) (Figure 2B), immobility duration (F5,38 = 5.19, *p* < 0.001) (Figure 2C), distance traveled (F5,38 = 5.78, *p* < 0.001) (Figure 2D), and average speed overall (F5,38 = 6.60, *p* < 0.001) (Figure 2E). Both doses of JMV2959 administered alone/with the vehicle did not cause significant changes in rat behavior during the monitored period in comparison to the vehicle + saline group.

### 2.3. JMV2959 and Ghrelin Effects on WIN55,212-2 Intravenous Self-Administration (IVSA)

**Figure 3:**
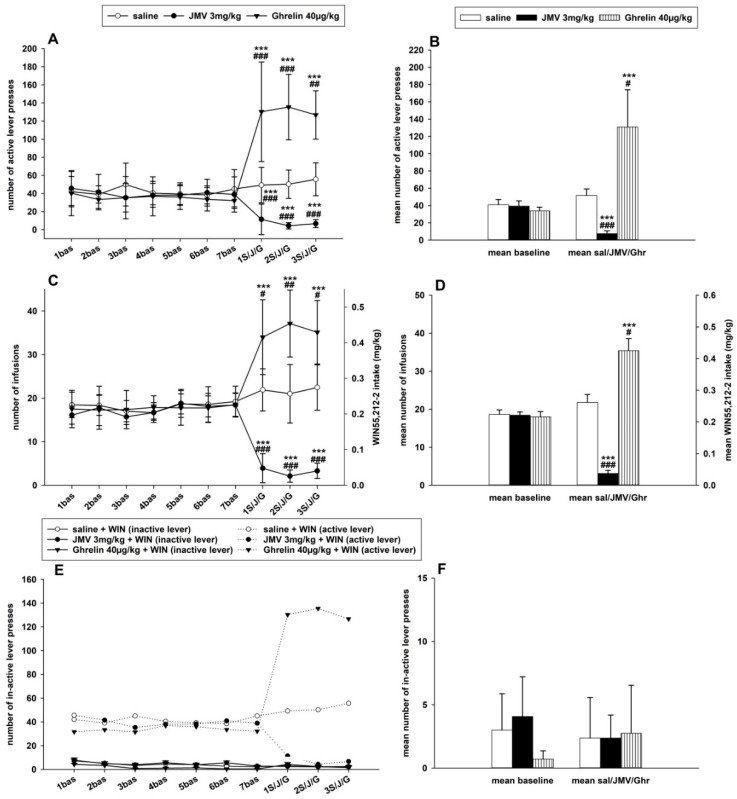
Effects of JMV2959 and ghrelin on WIN55,212-2 intravenous self-administration (IVSA). Saline (1 mL/kg) or JMV2959 (3 mg/kg) or ghrelin (40 µg/kg) were administered intraperitoneally 20 min before the 120 min IVSA sessions. Illustrated in graphs **A**,**C**,**E** are the daily active lever-pressings (**A**), number of infusions (**C**), and numbers of inactive lever-pressings (**E**) during the last week before pretreatments (1.–7. bas) and during three days of pretreatments (1.–3. S/J/G). Only the last three baselines (5.–7. bas) were used for statistical analysis by two-way repeated measures ANOVA followed by the Bonferroni test. The IVSA data went through logarithmic transformation before the statistical analysis; thus, in the graphs are presented original data together with significances obtained from the transformed ANOVA results. In graphs **B**, **D**, **F**, the means of saline/JMV2959/ghrelin (1.–3. S/J/G) active lever-pressing (**B**), infusions (**D**), and inactive lever-pressing (**F**) are illustrated together with the baseline means (5.–7. bas). The effects are presented as follows: Saline (open circle, open bar) (*n* = 9), JMV2959 (filled circle, filled bar) (*n* = 10), ghrelin (filled triangle, striped bar) (*n* = 8). Differences between the groups in comparison to saline group are expressed as # *p* < 0.05, ## *p* < 0.01, ### *p* < 0.001. Differences in the respective baseline mean within the group are expressed as *** *p* < 0.001. The results in graphs **A**,**C**,**E** are presented as group means with 95% confidence intervals. The results in graphs **B**,**D**,**F** are presented as means ± SEM.

In the main IVSA study, only rats with a minimum of 14 infusions per session during the baseline period were used for pretreatments and statistical evaluation. All rats were handled daily, controlled, and their weight was monitored during the whole IVSA experiment. The IVSA experiments were conducted during the reverse/dark period of a 12 h light/dark cycle. The last three 120 min self-administration sessions from a total of about 14 sessions prior to the pretreatments were used as baseline data. The *t*-test revealed significant differences between baseline active and inactive lever-pressing t(160) = 17.13, *p* < 0.001. Distinct inter-individual differences were observed within the basal WIN55,212-2 self-administration among all rats; however, the cannabinoid IVSA did not significantly differ among the groups that were pretreated with saline (*n* = 10), JMV2959 (*n* = 9), or ghrelin (*n* = 8) in all observed parameters: number of active and inactive lever-pressing, number of infusions, and WIN55,212-2 consumptions. The IVSA data failed the Lilliefors normality test and therefore went through logarithmic transformation before the statistical analysis (see the Section 4.6). Thus, in the figure, the graphs present the original results together with significances obtained from the ANOVA/Bonferroni tests with transformed data. Pretreatment with the GHS-R1A antagonist before all three consequent 120-min sessions significantly reduced the achieved basal maintenance WIN55,212-2 self-administration, while ghrelin pretreatment increased it (see Figure 3).

The active lever-pressing for WIN55,212-2 (demonstrated in Figure 3A,B) was always significantly attenuated when the GHS-R1A antagonist (JMV2959 3 mg/kg) was administered 20 min before the three consequent 2-h sessions in comparison to the saline group as well as to the baseline mean (*p* < 0.001). The two-way repeated measures ANOVA followed by the Bonferroni’s post hoc test, used for analyzing the JMV2959, ghrelin, and saline effects together, considering modulation of the same GHS-R1A receptor, revealed an overall significant effect of time (F1,150 = 15.55; *p* < 0.001) and the group × time effect (F2,150 = 5.03; *p* < 0.01), and the effect of group was not significant (F2,1 = 2.50; n.s.). The representative basal active lever-pressing (mean of five to seven baselines), which was used for statistical analysis, was 40.9 ± 6.1 in the saline and 39.3 ± 6.1 in the JMV2959 groups. Pretreatment with JMV2959 reduced the basal pressing to 7.4 ± 3.3 (mean of three pretreatment sessions), which represented 16.3% ± 4.4 of the baseline mean. The saline pretreatment resulted in 51.7 ± 7.6 (128.9% ± 7.8 of baseline mean) (the change was not significant in comparison to baseline mean). Pretreatment with ghrelin 40 µg/kg i.p. (20 min before sessions) increased the active lever-pressing in all rats in comparison to the baseline mean 33.9 ± 4.3 up to an average of 130.8 ± 43.2 (343.2% ± 77.5 of the baseline mean); however, extreme inter-individual differences were observed in response to ghrelin among the rats from a minimum of 151% to a maximum of 716% of the baseline. The variability of ghrelin-induced effects is further discussed in the Supplementary Information, where the daily active lever-pressing in single rats was illustrated as a percentage of the baseline mean (see Figure S2). The ghrelin-induced increase of active lever-pressing was significant in comparison to the baseline mean (*p* < 0.001) and also to the saline group with *p* < 0.001 during the first and second pretreatment and *p* < 0.01 in the third pretreatment session. The significant ghrelin-induced increase of active lever-pressing in comparison to the baseline mean (*p* < 0.001) and to the saline group (*p* < 0.05) is apparent also in Figure 3B, which illustrates comparisons of baseline and pretreatment means.

The number of infusions and the daily 2-h WIN55,212-2 intake/doses in mg/kg are illustrated in Figure 3C and the comparison of the average basal (5–7. baseline) and mean pretreatment (1–3. pretreatment) results is presented in Figure 3D. A 12.5-µg/kg/infusion dose of WIN55,212-2 was used in the FR1 self-administration schedule and each infusion was followed by 15-s time-out period; the active lever-pressing during the time-out was not rewarded. The body weights of the rats were rather homogenous during the IVSA experiment (see Table S1 in the Supplementary Information). The average basal number of infusions and WIN55,212-2 intake (mean of five to seven baselines) was 18.6 ± 1.2 infusions and 0.235 ± 0.015 mg/kg within the saline group and 18.4 ± 0.9 infusions and 0.230 ± 0.012 mg/kg in the JMV2959 group. Pretreatment with JMV2959 significantly (*p* < 0.001) reduced the number of infusions/consumptions of WIN55,212-2 to 16.4% ± 4.2 of the baseline mean, while after the saline pretreatment, the number of infusions/WIN55,212-2 intake reached 115.7% ± 6.3 of the baseline mean (which was not significant in comparison to the baseline mean). The two-way repeated measures ANOVA followed by the Bonferroni test analyzing the JMV2959, ghrelin, and saline effects together revealed overall significant differences among the groups (F2,1 = 7.78; *p* < 0.001), the group × time (F2,150 = 5.04; *p* < 0.01), and the effect of time (F1,150 = 49.23; *p* < 0.001). Pretreatment with JMV2959 always significantly (*p* < 0.001) reduced the number of infusions and the spontaneous WIN55,212-2 consumption also in comparison to the saline group. Pretreatment with ghrelin almost doubled the number of infusions and relevant WIN55,212-2 intake from basal values 18.0 ± 1.4 infusions and 0.225 ± 0.017 mg/kg to 35.4 ± 3.1 infusions and 0.443 ± 0.039 mg/kg, respectively (199.8% ± 17.8 of baseline mean), which represented a significant increase in comparison to the baseline (*p* < 0.001) as well as to the saline group (*p* < 0.01 in the second pretreatment and *p* < 0.05 within the other pretreatments). Similar to the active lever-pressing, the ghrelin-induced increase of the number of infusions/WIN55,212-2 intake was significant relative to the baseline mean (*p* < 0.001) and to saline group (*p* < 0.05) when the baseline and pretreatment means were compared (see Figure 3D).

The inactive lever-pressing, illustrated in Figure 3E,F, showed low basal activity (mean of five to seven baselines): 4.07 ± 3.1 in the JMV2959 group, 3.00 ± 2.9 in the saline group, and 0.71 ± 0.65 in the ghrelin group, and pretreatments did not produce any significant changes in all the analyses using the two-way repeated measures ANOVA followed by the Bonferroni test. 

**Figure 4:**
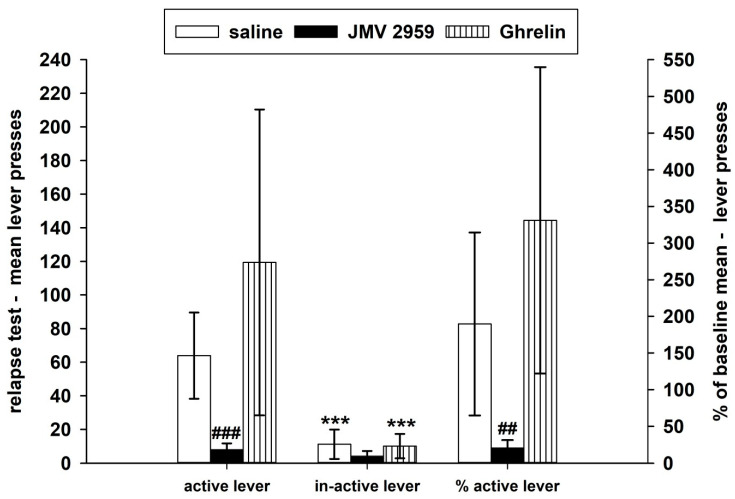
Effects of JMV2959 and ghrelin on WIN55,212-2-seeking lever-pressing/relapse-like behavior, observed on the twelfth day of forced abstinence of the WIN55,212-2 intravenous self-administration (IVSA) in active/inactive lever-pressing and percentage of the baseline mean (mean of the last three baselines before pretreatments, 5.-7. bas). Saline (1 mL/kg) or JMV2959 (3 mg/kg) or ghrelin (40 µg/kg) were administered intraperitoneally 20 min before the 120-min session, when the rats were in the IVSA cages not connected with the infusion pump. The IVSA relapse-test data went through logarithmic transformation before the statistical analysis; thus, in the graphs are presented original data together with significances obtained from the transformed ANOVA results. However, the percentage data were analyzed directly/not transformed using the Kruskal–Wallis one-way analysis followed by a Dunn´s test. The means of active lever-pressing in the groups are presented as follows: Saline (open bar) (*n* = 9), JMV2959 (filled bar) (*n* = 10), ghrelin (striped bar) (*n* = 8). Differences between the groups in comparison to the saline group are expressed as ## *p* < 0.001, ### *p* < 0.01. Differences between active and inactive lever-pressing are expressed as *** *p* < 0.001. The results are presented as group means with 95% confidence intervals.

During the forced abstinence, the rats were single housed in their home cages and were monitored daily. On the twelfth day of forced abstinence, the cannabinoid/WIN55,212-2-seeking/relapse-like behavior was tested back in the IVSA cages within a two-hour session under the standard IVSA conditions; however, the rats were not connected to the infusion pump. Thus, the active lever-pressing was not rewarded, it was only recorded as well as the inactive lever-pressing, as it is illustrated in Figure 4. Twenty minutes before the relapse-test session, JMV2959 or ghrelin or saline were administered to the appropriate animals. The two-way repeated measures ANOVA followed by the Bonferroni test (using the transformed data) was used for comparison of the active/inactive lever-pressing and the JMV2959/saline/ghrelin pretreatment effects (group), and it revealed significant differences among the groups (F2,1 = 16.80; *p* < 0.001), the type of lever-pressing (F1,48 = 45.21; *p* = 0.001), and the group × lever-pressing type effect (F2,48 = 5.01; *p* = 0.05). The WIN55,212-2-seeking behavior was significantly decreased by the JMV2959 pretreatment (*p* < 0.001) in comparison to the saline-pretreated group. After the ghrelin pretreatment the relapse-like behavior was increased, however, the difference was not significant in comparison to the saline-pretreated group. When the WIN55,212-2-seeking active lever-pressing was expressed in a percentage to the baseline-pressing mean (see Figure 4), a decrease to 20.6% ± 4.5 within the JMV2959 group, an increase to 189.6% ± 52.6 within the saline group, and a distinct increase to 330.9% ± 88.2 in the ghrelin-pretreated group were observed. The Kruskal–Wallis one-way analysis followed by the Dunn’s test comparison of active lever-pressing expressed in the percentage of baseline means during the relapse-test session (using original/not transformed data) revealed significant differences among the saline/JMV2959/ghrelin groups (H = 19.30 with 2 degrees of freedom; *p* < 0.001), specifically with the significant difference only between the saline and the JMV2959 groups (*p* < 0.01). The inactive lever-pressing was not expressed in a percentage because of zero occurring within the basal pressing. The apparent individual differences in reactivity of the rats to the appropriate pretreatments during the IVSA experiments including the relapse-test session are illustrated in Figure S2 within the Supplementary Information.

The rats received 20 g/d of standard chow food and ad libitum water throughout the IVSA conditioning and tests, and the daily food amount was always fully consumed by all rats regardless of any treatments. In the IVSA study, the body mass of the rats was measured daily, and no significant changes were observed concerning JMV2959 or ghrelin administrations (see Table S1 in the Supplementary Information).

### 2.4. JMV2959 and Ghrelin Effects on Vehicle and WIN55,212-2 Intravenous Self-Administration (IVSA) in an Additional Study

**Figure 5:**
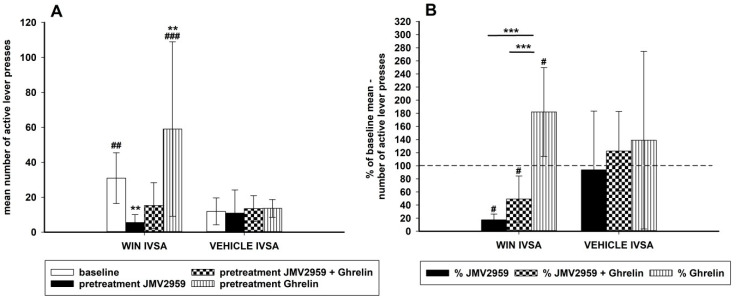
Additional IVSA experiment: Effects of JMV2959 and ghrelin on active lever-pressing for vehicle and for WIN55,212-2 are illustrated in graph (**A**). Baseline pressing (mean of three sessions before pretreatment) was influenced by pretreatment with JMV2959 (3 mg/kg) or JMV2959 + ghrelin or ghrelin (40 µg/kg) administered intraperitoneally 20 min before the 120-min sessions. The means of the active lever-pressing are presented as follows: basal lever-pressing (open bar), JMV2959 (filled bar), JMV2959 + ghrelin (dotted bar), ghrelin (striped bar). Differences between WIN55,212-2 IVSA and vehicle IVSA are expressed as ## *p* < 0.01, ### *p* < 0.001. Differences of pretreatments to baseline lever-pressing are expressed as ** *p* < 0.01. The effects of pretreatments illustrated in the percentage of the average baseline active lever-pressing (graph **B**) are presented as follows: percentage JMV2959 effect (filled bar), percentage JMV2959 + ghrelin effect (dotted bar), percentage ghrelin effect (striped bar). Differences between WIN55,212-2 IVSA and vehicle IVSA are expressed as # *p* < 0.05. Differences between pretreatments are expressed as *** *p* < 0.001. Dotted line shows the baseline active lever-pressing (100%). The additional IVSA data went through logarithmic transformation before the statistical analysis; thus, in the graphs are presented original data together with significances obtained from the transformed ANOVA results. The results are presented as group means with 95% confidence intervals (*n* = 4).

A separate group of rats was used in the additional IVSA for comparison of WIN55,212-2 IVSA with intravenous self-administration of the vehicle and the appropriate pretreatments, which is illustrated in Figure 5 in changes of active lever-pressing. Four rats self-administered the vehicle, another four the WIN55,212-2 again in a dose 12.5 µg/kg/infusion. Here the rats were chosen randomly with no demand for the minimum 14 daily infusions and other criterions; the IVSA arrangement was the same as in the main experiment (120-min sessions with schedule FR1, 15-s time-out, lights, etc.). The experimental schedule was as follows: the last three baseline 120-min sessions before pretreatments (from total 14 sessions) served as baseline values, then JMV2959 (3 mg/kg i.p.) was administered 20 min before two consequent sessions, before the third pretreatment session ghrelin (40 µg/kg i.p.) was applied together with JMV2959 (in separate injections), and then ghrelin (40 µg/kg i.p.) alone was injected 20 min before two consequent sessions. The *t*-test comparing all baseline data (three baselines before pretreatments) revealed significant differences between the WIN55,212-2 (18.0 ± 1.9) and vehicle (6.5 ± 1.1) number of infusions t(22) = 4.62, *p* < 0.001, as well as the number of active lever presses (30.0 ± 4.6 versus 11.9 ± 2.4) (t(22) = 4.62, *p* < 0.001). Active versus inactive lever-pressing was significantly different within the WIN55,212-2 IVSA (t(22) = 6.79; *p* < 0.001) (basal inactive lever-pressing 4.1 ± 1.0) and also within the vehicle IVSA (t(22) = 4.07, *p* < 0.01) (basal inactive lever-pressing 4.2 ± 1.2), but there were no significant differences within inactive lever-pressing either after pretreatments, nor between the IVSA cannabinoid/vehicle groups. Comparison of the number of active lever-pressing using the two-way repeated measures ANOVA followed by the Bonferroni test with factors IVSA type (WIN55.212-2/vehicle) (group) and pretreatments (baseline/JMV2959/JMV2959+ghrelin/ghrelin) (time) revealed significant differences among the groups (F1,6 = 3,87; *p* < 0.05), effect of time (F3,18 = 18.49; *p* < 0.001), and the group × time effect (F3,18 = 9.03; *p* < 0.001). However, the pretreatments had no significant influence on the vehicle IVSA. Within the cannabinoid IVSA, a significant reduction of active lever-pressing was observed after JMV2959 pretreatment to 17.4% ± 2.8 of baseline mean (*p* < 0.01 in comparison to baseline). This JMV2959 effect was attenuated by ghrelin co-administration during the third pretreatment session to 49.2% ± 11.0 (n.s. to baseline) and ghrelin pretreatment increased the active lever-pressing to 182.4% ± 21.3 (*p* < 0.01 to baseline). When the changes were expressed in the percentage of the baseline mean (see Figure 5B), the two-way ANOVA RM/Bonferroni confirmed the significant pretreatment effects within the WIN55,212-2 IVSA groups and no significant effects within the vehicle IVSA groups. The JMV2959, co-administration JMV2959 + ghrelin, and ghrelin pretreatment percentage changes were significantly different between the WIN55,212-2 and vehicle IVSA (*p* < 0.05) (effect of time F2,12 = 20.62, *p* < 0.001; group × time effect F2,12 = 10.57, *p* = 0.002; the effect of group was not significant, F1,6 = 1.19). Observed changes in the number of infusions were similar and are illustrated and described in the Supplementary Information (Figure S3). 

## 3. Discussion

To our knowledge, our results demonstrated for the first time that GHS-R1A antagonism significantly reduced cannabinoid/WIN55,212-2 intravenous self-administration (IVSA) and suggested reduction of the cannabinoid-seeking/relapse-like behavior, reduced behavioral stimulation and development, as well as expression of the cannabinoid/THC-induced conditioned place preference (CPP).

The CPP method mainly studies the association and conditioning of environmental cues with the drug effect, which play an important role in the acquisition and maintenance of addiction [47]. Cannabinoids, including tetrahydrocannabinol/THC, are known for their general biphasic/dual effects [48]. Lower THC doses (around 0.1–0.3 mg/kg) are linked with rewarding and stimulatory effects, while higher doses (1 mg/kg and greater) produce hypoactivity and aversion [49]. In accordance with the literature, also in our present rat study the conditioning with THC 0.3 mg/kg i.p. induced the CPP. The GHS-R1A antagonist (1 and 3 mg/kg JMV2959) that was administered together with THC during conditioning dose-dependently reduced the development of (biased) CPP; but only the higher dose induced a highly significant effect (*p* < 0.001). It has been well established that cannabinoids support CPP through activation of CB1 receptors since the antagonist (SR141716A) can reverse this effect [50,51]. The rewarding/reinforcing effects of cannabinoids/THC are most likely mediated through mesolimbic CB1 receptors via dopamine release trigger within the nucleus accumbens (NAC), similarly to other drugs of abuse [4,19,20,52]. Previously, it has been described that JMV2959 pretreatment significantly reduced or abolished dopamine efflux induced by alcohol [44], stimulants (cocaine, nicotine, and amphetamine) [53,54], and opioids [29,30,55] in rodents. In our recent experiments, JMV2959 also reduced the WIN55,212-2-induced accumbens dopamine release (published at this Special Issue [33]). Thus, we can presume that the THC rewarding/reinforcing effects were at least partly reduced by simultaneous GHS-R1A antagonism of dopamine release during conditioning, which consequently decreased the CPP development.

Single administration of 1 and 3 mg/kg JMV2959 dose-dependently and significantly (*p* < 0.001) reduced the THC-CPP expression. Thus, JMV2959 significantly reduced the manifestation of the developed place conditioning with THC experiences which suggests that the GHS-R1A antagonism attenuated the anticipation of the previously retained reward, an attribute of craving. This corresponds with studies in mice/rats, when ghrelin antagonism reduced expression of CPP induced by alcohol [44], stimulants [53,54,56], and opioids [28,29,55]. Previous studies documented that JMV2959 alone did not induce the CPP [44] or conditioned taste aversion [57]. Our previous experiments suggested that JMV2959 alone (1, 3, and 6 mg/kg) did not significantly influence the rat locomotion in the activity cage, when monitored within 25 to 45 min after JMV2959 administration [28].

Most drugs of abuse tend to induce behavioral stimulation, which is considered to be a sign of the nigrostriatal dopaminergic pathway activation, which may become sensitized and contribute to drug addiction [45,58]. In accordance with the above-mentioned biphasic character of effects, it was described that low cannabinoid/THC doses increase ambulation and rearing in rodents, which can be most clearly observed after certain habituation period, when the initial explorative activity of animals subsided and the cannabinoid advanced stimulatory effects were unmasked [45,46]. Identically, in our LABORAS cage experiment, 0.1 mg/kg THC significantly increased locomotion, rearing, distance traveled, and the overall average speed of behavior in comparison to the control/vehicle treated group (within 20–40 min after THC/vehicle administration). Pretreatment with JMV2959 (1 and 3 mg/kg), dose-dependently and significantly reduced all the monitored hyperactivity parameters (*p* < 0.001). These results correspond with studies in mice/rats, when JMV2959 attenuated behavioral stimulation induced by alcohol [44], cocaine and amphetamine [53], nicotine [54], morphine [30,55], and fentanyl [32], as well as WIN55,212-2, which was documented in our recently published study [33]. The same JMV2959 doses which were administered alone did not significantly influence the rat behavior within the appropriate period in the LABORAS cage, which was in accordance with our previous study in activity cage/open field [28].

The IVSA method, a crucial experimental model for addiction research, enabled us to estimate the drug rewarding/reinforcing abilities and evaluate the principal treatment goal of reducing or abolishing the drug-taking behavior. Despite clear evidence of the addictive potential of cannabis use in humans [4,19], the utilization of the IVSA model in cannabis/cannabinoids research appears to be rather ambiguous. A convincing THC self-administration was observed in nonhuman primates [59], but robust THC IVSA in rodents has not been reported [37,60]. Perhaps a combined IVSA of THC together with cannabidiol/CBD induced reliable intake in rats [61]. However, intracerebroventricular (i.c.v.) self-administration of THC was demonstrated in rats, similarly with the bicyclic synthetic cannabinoid CP55940 [62,63]. Furthermore, IVSA of aminoalkylindole cannabinoid WIN55,212-2 was established in rats and mice [36,37,38,64]. The substantial role of the CB1R in the cannabinoid reinforcing effects was supported by the reversal of self-administration with the CB1 receptor antagonist rimonabant [36,37,62,63]. The THC is considered as a partial CB1R and CB2R agonist [4,43], while WIN55.212-2 acts as a full CB1R/CB2R agonist [35] as many further synthetic cannabinoids extensively abused in Europe within last years [1]. In a recent literary study, THC failed to maintain IVSA in WIN55,212-2 self-administering/trained rats and the responsible factors are yet to be clarified [37]. Thus, the WIN55,212-2 IVSA rat model is fully suitable for testing of the general cannabinoid/CB1R agonist reinforcing effects and the involved mechanisms (such as possible GHS-R1A involvement). Thus, it might help to suggest mechanisms potentially reducing the CB1R-agonist drug-taking behavior, but the feasible transferability of the obtained results to cannabis (marihuana, hashish) use has distinct limits [37].

Taking into consideration the dual/biphasic effect of cannabinoids, a WIN55,212-2 dose of 12.5 µg/kg/infusion was chosen, which according to the literature had the most reinforcing effects. During the maintenance period, our WIN55,212-2 IVSA studies were in accordance with the literature [36,37,38]. The inactive lever-pressing was significantly lower than the active lever-pressing. Moreover, the vehicle (saline with a drop of Tween 80) IVSA was significantly lower than the WIN55,212-2 IVSA in both of our studies, which adequately confirmed the reinforcing effects of the cannabinoid [4,19,37]. Pretreatment with the GHS-R1A antagonist significantly reduced the basal maintenance WIN55,212-2 IVSA in both studies and all monitored parameters: the number of active lever-pressing, number of infusions, and daily consumptions in mg/kg (the inactive lever-pressing was mainly not significantly influenced). Pretreatment with JMV2959 (3 mg/kg i.p.) reduced the basal WIN55,212-2 IVSA in the main study to average 16.4% ± 4.2 (infusions) and 16.3% ± 4.4 (active lever-pressing), and in the additional study to average 23.9% ± 5.0 (infusions) and 17.4% ± 2.8 (active lever-pressing). The cannabinoid self-administration was completely abolished in three sessions (in two different rats), and in nine sessions the rats produced only one infusion. This suggests that the GHS-R1A antagonist markedly reduced the WIN55,212-2/cannabinoid-induced reinforcing/rewarding effects. Furthermore, JMV2959 pretreatment also significantly reduced the WIN55,212-2-seeking/relapse-like behavior tested in the IVSA cage on the twelfth day of forced abstinence, when the non-reinforced active lever-pressing decreased to 20.6% ± 4.5 of the baseline mean. Within the saline group, the non-reinforced active lever-pressing during the relapse-test session achieved a 189.6% ± 52.6 of the baseline, which indicates the incubation of the cannabinoid craving, similarly to other previous studies [65]. In our experimental schedule, the same animals were pretreated with JMV2959/ghrelin during the maintenance IVSA period and during the relapse-test session. Thus, it should be taken into consideration that the previous pretreatment history might have influenced the rat behavior during the drug-seeking session. Further experiments with absent pretreatments during the maintenance IVSA would more precisely document the GHS-R1A antagonism effectiveness specifically on the cannabinoid-seeking behavior. Our WIN55,212-2 IVSA results are in accordance with the few known self-administration studies dealing with GHS-R1A-antagonism in the alcohol, sucrose [66,67,68,69], fentanyl [29], and methamphetamine [56] rodent models. 

However, JMV2959 did not influence the vehicle IVSA. These results are consistent with other published findings, when the JMV2959/GHS-R1A antagonism significantly reduced reinforcing effects, such as ghrelin/hexarelin-provoked food intake, increased weight gain and fat mass, the sucrose self-administration, and consumption of rewarding food [26,68]. However, when JMV2959 was administered alone, it did not significantly influence the standard food consumption and body mass in rodents [26,68,70], or the locomotor activity or the accumbens dopamine in rats/mice [28,30,44,55]. In addition, in our present IVSA study, the JMV2959 treatments did not affect the rat body mass.

On the contrary, administration of ghrelin (40 µg/kg i.p.) significantly increased the number of infusions and active lever-pressing to 199.8% ± 17.8 and 343.2% ± 77.5 of the baseline mean, respectively. The observed noticeable inter-individual differences in the rats’ active lever-pressing after the ghrelin pretreatment (from minimum 151% to maximum 716% of baseline mean) indicated heterogenous sensitivity of the rats to the ghrelin-increasing effect on motivation to the cannabinoid self-administration. In addition, ghrelin pretreatment during the relapse-test session augmented the non-reinforced cannabinoid-seeking active lever-pressing to 330.9% ± 88.2 of the baseline mean and the active lever-pressing tended to be higher in comparison to the saline group. However, as mentioned above, the craving incubation during the abstinence period had increased the active lever-pressing within the saline group. In addition, values within the ghrelin group were rather spread (110–642% of the baseline mean) similarly to the saline group (77–565% of the baseline mean); thus, the comparison between the saline and ghrelin groups did not reach statistical significance in the relapse-test session. These results suggest that ghrelin supported/enhanced the cannabinoid´s attraction for rats and their motivation for active lever-pressing. This is in accordance with the literature, when intracerebral administration of ghrelin increased alcohol intake [44] and heroin IVSA [71] and peripheral administration of ghrelin increased cocaine-induced potentiation of alcohol consumption [72] in rats. Simultaneously, ghrelin did not affect the vehicle IVSA. Previously, it was described that ghrelin alone did not influence locomotion in rats [73]; however, it increased not only the palatable food and sucrose consumption but also the freely available chow [74,75]. It should be taken into consideration that ghrelin has its own reinforcing properties [76,77]. In our additional IVSA study, ghrelin co-administration together with JMV2959 abolished the significant JMV2959-induced attenuation of WIN55,212-2 IVSA in the active lever-pressing parameter (from *p* < 0.01 to n.s. in comparison to baseline) and also in the number of infusions (from *p* < 0.001 to n.s. relatively to baseline). This suggested the involvement of the GHS-R1A mechanisms. However, it should be mentioned that the principal aim of the additional IVSA arrangement was to compare the vehicle and WIN55,212-2 self-administration and to monitor the JMV2959 effects in the control vehicle IVSA conditions. In our continuous non-randomized experimental schedule within the additional IVSA, the observed effects might have been affected by the previous treatment history; thus, the presented results should be considered with certain limitations. For a more specific investigation of the pretreatment effects on the WIN55,212-2 IVSA, the employment of a randomized schedule or prolonged free session intervals between pretreatments would be far more appropriate. Nevertheless, even in our indicative experiment, when the JMV2959 combination with ghrelin was used as an interface between the alone JMV2959 and alone ghrelin sessions, the effect of the co-administration was noticeable.

Altogether, our IVSA results demonstrated the important involvement of ghrelin/GHS-R1A in the rewarding/reinforcing effects of WIN55,212-2, which complements our behavioral studies with THC (CPP and LABORAS); thus, there is strong indication that the central ghrelin system crucially participates in the rewarding/reinforcing pro-addictive effects of cannabinoids similarly to alcohol, stimulants, and opioids [10,11,13]. Certainly, further research of potential employment of the GHS-R1A antagonism to reduce signs of cannabinoid addiction behavior should carefully consider the usual mode of cannabinoid administration (inhalation), the distinct differences among the cannabinoid types, the particularities of cannabis, and other factors.

As it has been already mentioned, the cannabinoids (including THC and WIN55,212-2) through mesolimbic CB1Rs increase the dopamine concentration in the NAC followed by further reinforcement, conditioning, and salience alteration processing [4,48,78,79,80,81]. Furthermore, the ghrelin antagonism was observed to decrease accumbens’ dopamine efflux induced by alcohol, stimulants, and opioids (see above and the reviews, [10,11,13]. In our recent study, we documented that JMV2959 pretreatment also reduced the WIN55,212-2-induced accumbens dopamine release (published in this Special Issue [33]). Thus, we can assume that JMV2959 pretreatment reduced the observed cannabinoid/WIN55,212-2/THC rewarding/reinforcing effects at least partly by GHS-R1A antagonism of accumbens dopamine release. However, the involved mechanisms of action are probably more complex. Ghrelin/GHS-R1A has comprehensive interrelationships with a multitude of other systems [7,32,82]. In addition to the distinct constitutive activity [83,84,85], GHS-R1A oligomerizations and dimerizations have been found with a wide array of other G-protein coupled receptors, etc. [82]. Important mutual interactions between endocannabinoids and ghrelin participating in hedonic food intake have been confirmed [22,23,86]. Further research is necessary to clarify the involved mechanism.

Our results demonstrated that GHS-R1A plays a significant role in the THC/WIN55,212-2/cannabinoid rewarding/reinforcing effects, which encourages further research of the GHS-R1A antagonism as a potential approach to cannabinoid addiction treatment. 

## 4. Materials and Methods

### 4.1. Animals

Male adult Wistar rats (Velaz, Prague, Czech Republic) initially aged about 8 weeks were used in all experiments. At least seven days before the beginning of the experiments, the rats were given free access to food and water and they were housed in polycarbonate cages with a constant humidity (50–60%) and room temperature (22–24 °C). The conditions were also the same between the experimental procedures, with the exception of the rats throughout the IVSA conditioning and tests, when they received a 20 g/d standard chow food and ad libitum water. In our studies, the food was always removed (if it was not consumed) following any drug administration within running experiments. The rats in the conditioned place preference (CPP) experiment were housed in a normal 12-h light/dark cycle (6 a.m.–6 p.m.), and animals included in the intravenous self-administration (IVSA) and LABORAS experiments in a reverse 12-h light/dark cycle. The rats were accommodated individually (IVSA), or 3 in one cage (CPP, LABORAS). The rats in the IVSA study were handled daily prior to experiments to get familiar and less stressed during the procedures. Procedures involving animals, along with animal care, were conducted in accordance with international laws. The protocols complied with the Guidelines of the European Union Council (86/609/EU, 24 November 1986) and the EU Directive (2010/63/EU, 22 September 2010), and followed the instructions of the National Committee for the Care and Use of Laboratory Animals. All experiments were under the Expert Committee for Protection of Experimental Animals of the Third Faculty of Medicine, Charles University in Prague, and they were performed in accordance with the Animal Protection Act of the Czech Republic (No. 246/1992 Sb, 15 April 1992).

### 4.2. Drugs and Chemicals

Tetrahydrocannabinol (THC) was synthesized in cooperation with the University of Chemistry and Technology Prague (UCT Prague, Czech Republic), and the synthetic aminoalkylindole cannabinoid WIN 55,212-2 mesylate salt (WIN55,212-2) was provided by Sigma–Aldrich (Prague, Czech Republic). Ghrelin was purchased from Essence Line (Prague, Czech Republic). The GHS-R1A antagonist, substance JMV2959 (1,2,4-triazole derivate) [26], was synthetized by the UCT Prague (Czech Republic). Both THC and WIN55,212-2 were firstly dissolved in one drop of Polysorbate 80 (Tween 80) and then diluted in saline. Instead of THC/WIN55,212-2 as the vehicle (saline with one drop of Tween 80) and instead of JMV2959/ghrelin pretreatments, saline served as the placebo/control. THC was used in a stimulatory/rewarding 0.1 mg/kg dose in LABORAS, and rewarding 0.3 mg/kg dose in CPP, in accordance with the literature [45,46], and administered intraperitoneally (i.p.) in volumes of 0.1 mL/100 g of body weight. It has been described that, in comparison with THC, WIN55,212-2 has been reliably self-administered in rodents/rats [36,37,38]; therefore WIN55,212-2 was used for intravenous self-administration in 12.5 μg/kg/infusion in volumes of 0.1 mL per infusion/active lever press. The selected doses of JMV2959 (1 or 3 mg/kg) were determined based on our previous studies in Wistar rats [29,56] and had no significant effect on the rat locomotor behavior [28]. JMV2959 was administered 20 min prior to the IVSA sessions, the THC/saline administrations in LABORAS and CPP testing trials or together with THC during the conditioning process during the second CPP experimental arrangement. Ghrelin was administered in dose 40 µg/kg i.p. 20 min prior to the IVSA sessions.

### 4.3. THC-Conditioned Place Preference (CPP)

The biased conditioned place preference (CPP) method was based on our previous experiences and the literature [28,29,53,56,87]. A three-compartment CPP apparatus, with distinct visual and tactile cues in the outer compartments was used with open-able/shut-able gates between the compartments. Whole apparatus was illuminated by 45 lux. The experiment consisted of pre-conditioning (day 1), conditioning (days 2–9), and post-conditioning (day 10). On day 1 (pre-conditioning), each rat was injected i.p. with saline 20 min prior testing, then placed in the central compartment with both gates open, and initial/spontaneous place preference was determined during the 20 min. Conditioning was performed using a repetitive procedure in which THC (0.3 mg/kg i.p.) was paired to the spontaneously least preferred compartment. In the first experimental arrangement, during the 8-day conditioning period, each rat received a total of two i.p. injections per day in a balanced design; THC was administered in the morning and saline in the afternoon and vice versa. After each drug injection, the rat was placed in the appropriate outer compartment (for 30 min, with the gate closed). On day 10 (post-conditioning test session), the rats were placed in the central compartment (with the gates open) and were given free access to both compartments for 20 min. To evaluate the effects of the GHS-R1A antagonist on the expression of THC-CPP, each rat was acutely injected with JMV2959 (1 or 3 mg/kg i.p.) or saline (i.p.) 20 min prior to the test session (number of rats in the groups *n* = 8–11). In the second experimental arrangement, the effects of GHS-R1A antagonism on the development of THC CPP were tested in a separate experiment, when JMV2959 (1 or 3 mg/kg i.p.) or saline (i.p.) were administered repeatedly during the 8-day conditioning phase, always together with THC in separate injections into different sites on the rat (*n* = 9–10). CPP was calculated as the difference in the percentage of the total time spent in the THC-paired (i.e., least spontaneously preferred) compartment during the post-conditioning and pre-conditioning sessions. Another mode of CPP calculation was used in the Supplementary Information in order to corroborate the findings (see Figure S1). It has been previously described that the application of the vehicle/saline as well as JMV2959 per se does not induce any CPP conditioning [44]; therefore, these experiments were not included.

### 4.4. Behavioral Testing in the LABORAS Cage

For behavioral testing, the LABORAS apparatus was used from 8 a.m. to 4 p.m. in a reversed light/dark cycle (during the dark period). The LABORAS is a fully automated system for continuous behavior recognition and tracking of small rodents [88]. Rats were placed into the LABORAS cage immediately after i.p. injection of saline or JMV2959 (1 or 3 mg/kg) for habituation, anf 20 min later 0.1 mg/kg THC or vehicle was administered i.p. and rats were left in the cage for another 20 min habituation. Then, the 20 min monitored period started; thus, the behavior changes were measured within 20–40 min after THC, when significant THC-induced behavioral stimulation could be observed [45]. The following parameters were automatically evaluated by LABORAS: Time spent in locomotion [s], time spent immobile [s], time spent rearing [s], time spent grooming [s], distance (trajectory length) [m], and average speed [mm/s]. The animals were randomly assigned to groups (*n* = 7–9, single time 4). The group administered with vehicle + saline was used as a control to compare the effects of THC and the pretreatments.

### 4.5. WIN55,212-2 Intravenous Self-Administration (IVSA)

**Figure 6:**
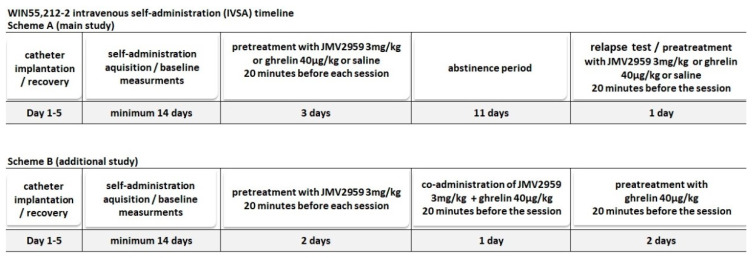
The time-line schedules of the IVSA experiments within the main IVSA study (**A**) and the additional IVSA study (**B**).

Forty-four naive male rats were used in this study; groups of 10 (JMV2959), 9 (saline group), and 8 (ghrelin group) were used in the statistical analyses in the main WIN55,212-2 IVSA study; four rats self-administered vehicle and a further four rats WIN55,212-2 in the additional IVSA experiment; seven rats were excluded because they did not reach the minimal daily cannabinoid intake (minimum 14 infusions), and two rats for leakage. Under ketamine–xylazine anesthesia (ketamine 100 mg/kg i.p., Narketan, Vetoquinol, France; xylazine 10 mg/kg i.p., Xylapan, Vetoquinol, France), rats were surgically implanted with a permanent intracardiac silastic catheter through the external jugular vein to the right atrium. The outer part of the catheter exited the skin in the midscapular area, and it was fixed in the needleless input (SAI Infusion Technologies, Lake Villa, IL, USA). After the catheter implantation surgery, the catheters were flushed with heparin (heparin sodium/Heparin Leciva, Zentiva), antibiotics (cefazoline/Cefazolin, Sandoz, Austria), and analgesics (meloxicam, Metacam, Boehringer Ingelheim/Rhein, Germany). The self-administration sessions started on the sixth day after surgery. The catheters were flushed with a cocktail of 0.3 mL of saline and heparin solution (5 IU) in order to prevent occlusion in the catheters and to assess the catheter’s patency before and after each self-administration session. Changes in general behavior, catheter patency, the body weight, and food intake of each animal were recorded daily. Experimental cages with two levers located on one side of the cage were programmed by Graphic State Notation 3.0.3. Software (Coulbourn Instruments, Whitehall, PA, USA), and the IVSA sessions were conducted under the fixed ratio schedule of reinforcement (FR1; each correct response reinforced). An active lever-pressing (combined with a cue light) led to the activation of the infusion pump and administration of a single infusion of WIN55,212-2 (dose 12.5 μg/kg/infusion/0.1 mL) followed by a 15-s time-out, while an inactive lever-pressing was recorded but not rewarded. The cue light was flashing during dose infusion and off during the time-out. The house light was also flashing during each infusion. The sessions lasted for 120 min and were performed twice daily (once daily for each animal) on working days. In the main IVSA study, we wanted to test the potential antagonistic effects of the GHS-R1A antagonist/JMV2959 in the reliable WIN55,212-2 self-administration model; thus, we chose adequate exclusion criterion which would guarantee a convincing level of self-administration. After a stabile drug consumption for at least seven sessions (above 70% preference of the active lever, minimum 14 infusions during a session) and after two consequent sessions with a maximal deviation of 15%, rats were pretreated with JMV2959 (3 mg/kg i.p.) or ghrelin (40 µg/kg i.p.) or saline (0.1 mL/100 g body weight i.p.) 20 min before the IVSA session for three consecutive days. The next day, the 11-day abstinence period started. During the abstinence period, animals were housed individually in their cages. On the twelfth day of abstinence, the rats were placed again into their IVSA cages for one session, and disconnected from the infusion pump, to test the cannabinoid-seeking/relapse-like behavior (the lever-pressing was monitored). Twenty minutes before this drug-seeking test session, the rats were again pretreated with JMV2959 (3 mg/kg) or ghrelin (40 µg/kg i.p.) or saline (0.1 mg/100 g). The experimental schedule of the main IVSA study is illustrated below in Figure 6A. The numbers of active and inactive lever-pressing, number of infusions, and WIN55,212-2 consumption (μg/kg) were statistically analyzed. The last three sessions/days with a stabilized WIN55,212-2 IVSA intake before pretreatment (5.–7. baseline), three consequent JMV2959/saline pretreatment sessions and “relapse-test” sessions were finally used in the statistical analysis. In the additional IVSA study, we wanted to document the WIN55,212-2 reinforcement effects in comparison to the vehicle IVSA and to test the pretreatment (JMV2959 and ghrelin) effects per se in the control vehicle IVSA conditions. Thus, the vehicle instead of cannabinoid was self-administered by four rats and WIN55,212-2 was self-administered by another four rats. After 14 days of IVSA (with no exclusive criterion), these rats were pretreated equally with JMV2959 (3 mg/kg i.p.) 20 min before the two consequent IVSA sessions; then, they were pretreated with JMV2959 (3 mg/kg i.p.) together with ghrelin (40 µg/kg i.p.) before the third pretreatment session and then they were pretreated again with only ghrelin (40 µg/kg i.p. 20 min before IVSA) for another two consequent sessions. In the main IVSA study, we observed slightly intensified pretreatment effects during the second pretreatment session; thus, we wanted to observe the effect of repeated JMV2959/ghrelin administration per se in the vehicle IVSA. The combination of the GHS-R1A antagonist/JMV2959 with GHS-R1A agonist/ghrelin should show the co-administration effect on the vehicle IVSA and try to prove the involvement of the GHS-R1A in the JMV2959 effects. Specifically, we wanted to test if co-administration with ghrelin would attenuate the JMV2959-induced reduction of the WIN55,212-2 IVSA. The co-administration was used as an interface between the single JMV2959 and ghrelin pretreatments. The experimental schedule of the additional IVSA study is illustrated below in Figure 6B. During the whole IVSA experiment, the body mass of all rats was monitored daily, and the difference between groups and possible impact of JMV2959 treatment on the body mass was statistically evaluated in the main IVSA study, within the last seven days before pretreatment, during the three days of pretreatment, the tested relapse-like behavior day, and during all evaluated periods (7 baselines + 3 pretreatment days + relapse-like behavior day = 11 days).

### 4.6. Statistical Analysis

Sigma Plot 13 (Systat Software, Inc., San Jose, CA, USA) was used for the statistical evaluation of the data from CPP and LABORAS and the R program (Lucent Technologies, Wienna, Austria; R Core Team 2013) was used for evaluation of the data from the IVSA study. The appropriate evaluated data (CPP, LABORAS) were subjected to the Shapiro–Wilk normality test and the IVSA data were subjected to Lilliefors test of normality (Kolmogorov–Smirnov test for not fully specified normal distribution). Homogeneity of variance for analysis of variance (ANOVA) was tested using Levene´s test. Place preference scores (CPP) were calculated as a difference in percentage (%) of total time spent in the THC-paired (spontaneously least preferred) compartment during the postconditioning and the preconditioning session. The evaluated CPP data passed the normality test. The equal variance test passed in the CPP arrangement when the JMV2959 was administered repeatedly during conditioning. The equal variance test passed when the acute JMV2959 was administered at the lower 1 mg/kg dose and it failed with the higher 3 mg/kg acute dose in comparison with the saline group. The differences between groups were evaluated by one-way ANOVA followed by the Holm–Sidak post-hoc test. In the Supplementary Information the CPP absolute values of time spent in the THC-paired compartment during the pre- and post-conditioning sessions were compared using the two-way repeated measure ANOVA followed by the Bonferroni´s post hoc test. The behavioral changes observed within 20–40 min after the THC/vehicle administration in the LABORAS cage, among the groups of rats with different treatments, were evaluated by one-way ANOVA followed by the Holm–Sidak post hoc test. The evaluated data from the LABORAS cage experiment passed the equal variance tests and the normality tests. The evaluated data obtained during the IVSA procedure (the corresponding numbers of active and inactive lever presses and number of infusions) failed the equal variance tests. In addition, when the Lilliefors test of normality was applied, the acceptable use of normal distribution was rejected for all rat groups in IVSA. The lognormal distribution was suggested as adequate; therefore, the logarithmic transformation (LN) of the data was used to fulfil normality. These transformed data were used for the statistical significance calculations. In the graphs, the original data are illustrated with the ANOVA/Bonferroni significances obtained using the transformed data. During the IVSA procedure, the comparison of active and inactive lever-pressing was conducted using a *t*-test within all analyzed baseline data of the last three sessions before pretreatments. In the main IVSA study, the statistical differences between the saline versus JMV2959 or ghrelin groups relative to time/session and procedure related changes were calculated by two-way repeated measures analysis of variance (ANOVA RM), with the group (saline/JMV2959/ghrelin) and time/session (5.–7. baselines, 1.–3. pretreatments) as factors, followed by Bonferroni post-hoc tests. The difference between groups in the lever-pressing during the WIN55,212-2-seeking/relapse-like behavior testing session, when the rats were not connected to the infusion pumps, was analyzed separately using the two-way ANOVA/Bonferroni with the group (saline/JMV2959/ghrelin) and lever-pressing type (active/inactive lever-pressing) as factors (again after logarithmic transformation of the data). When the drug-seeking active lever-pressing data were expressed in percentage of the baseline mean, the results again failed the normality test; therefore, the Kruskal–Wallis one-way analysis followed by the Dunn´s test was used for analysis of the differences among the groups (thus, these percentage data were not transformed). Except for the “relapse-test” session with no infusions, for each daily session, all IVSA parameters were calculated as a total number of active and inactive lever-presses, infusions, and WIN55,212-2 consumption (mg/kg) during the appropriate 2-h daily sessions. The last three sessions/days of the WIN55,212-2 IVSA prior to the pretreatments, the three pretreatment sessions (saline/JMV2959/ghrelin), and the relapse-test session were used in the statistical analyses. In the additional IVSA experiment, a two-way repeated measures ANOVA/Bonferroni test was used for comparison of the WIN55,212-2 and vehicle IVSA (group factor) and the time/pretreatment effects (non-pretreated baselines, two JMV2959, one JMV2959 + ghrelin, two ghrelin pretreatment sessions); means of the last three baselines before the pretreatment were used in the statistical analyses (mean of 5.–7. baseline sessions). Comparison of the WIN55,212-2 and vehicle lever-pressing was also conducted using a *t*-test within all baseline data of the last three sessions before pretreatments. All statistical tests were evaluated at a significance level of 0.05 (P-values of <0.05, <0.01, and <0.001 defined statistical significance). The average results (mean of 3 baselines, mean of 3 pretreatment sessions) within the main IVSA study were illustrated as means ± SEMs. All further results were presented as the group means with 95% confidence intervals (95% CI). The 95% CI modification for small groups was applied using the appropriate *t*-values for calculations. In the IVSA studies, the data failed the Lilliefors normality test and therefore went through logarithmic transformation before the statistical analysis (see above). Thus, the original results are presented in the figure graphs together with significances obtained from the ANOVA/Bonferroni tests with transformed data.

## Data Availability

The data presented in this study are available in the article and Supplementary Information.

## Supplementary Materials

Supplementary materials are available online at https://www.mdpi.com/1422-0067/22/5/2397/s1.
