# Supplementary material for: Cannabinoid-Induced Conditioned Place Preference, Intravenous Self-Administration, and Behavioral Stimulation Influenced by Ghrelin Receptor Antagonism in Rats
Source: Int J Mol Sci. 2021 Feb 27;22(5):2397. doi: 10.3390/ijms22052397 (PMC7957642; doi:10.3390/ijms22052397)
Supplement: Supplementary file 1 [file ijms-22-02397-s001.pdf]

SUPPLEMENTARY INFORMATION

RESULTS

JMV2959 effects on manifestation and development of THC-induced conditioned place preference (CPP)

In the main article, the effects of JMV2959 pretreatments on the THC- CPP are expressed as the difference in percentage of total time spent in the THC-paired (i.e., least spontaneously preferred) compartment during the post-conditioning (day 10) and/minus the pre-conditioning (day 1) session. Whereas, in the Figure S1, the results of the same CPP experiments are processed and illustrated in a different way: there are the absolute values of time spent in the THC-paired/least preferred compartment before (pre-conditioned session, day 1) and after conditioning (post-conditioned session, day 10). These results were statistically evaluated using two-way repeated measures ANOVA/Bonferroni, with the group/treatment (0, 1, 3 mg/kg JMV2959) and the time/session as the factors. In both CPP arrangements the THC-CPP was established ($p < 0.001$). The acute JMV2959 administration after the THC conditioning significantly and dose dependently reduced the THC-CPP expression ($p < 0.05$ and $p < 0.001$)(see Figure S1A). The two-way ANOVA RM/Bonferroni revealed an overall effect of the time ($F_{1,24} = 9.32$, $p = 0.005$) and the group x time interaction ($F_{2,24} = 8.65$, $p = 0.001$), the effect of group was not significant ($F_{2,24} = 1.70$, n.s.). The repeated JMV2959 administration with the THC during conditioning together significantly reduced the THC-CPP development only when the higher 3 mg/kg JMV2959 dose was used; the lower 1 mg/kg JMV2959 dose was not significant (see the Figure S1B). The two-way ANOVA RM/Bonferroni revealed overall effect of the group ($F_{2,25} = 4.51$, $p = 0.02$), the time ($F_{1,25} = 17.60$, $P < 0.001$) and the group x time interaction ($F_{2,25} = 9.52$, $p < 0.001$). Thus, these results match the findings illustrated in the main article (see Figure 1), when different mode of calculation was used.

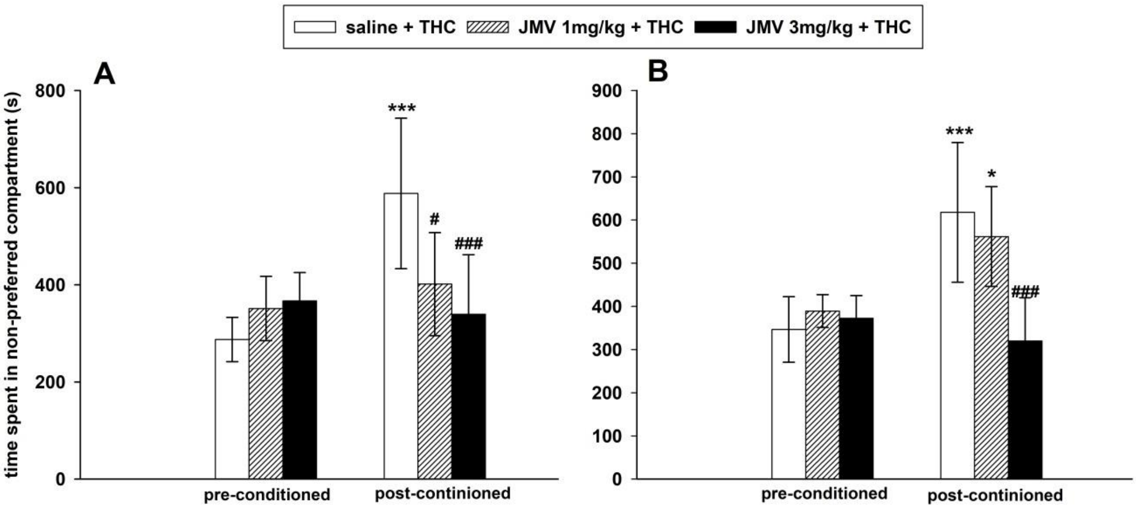

Figure S1: Effects of JMV2959 on the THC-CPP. The graphs show mean time spent by the rats in the THC-paired (thus spontaneously non-preferred) compartment before (pre-conditioned/day 1) and after 8 days of conditioning with THC 0.3 mg/kg (post-conditioned/ day 10). In the graph A, JMV2959 (0, 1, 3 mg/kg) was administrated in a single dose 20 min before final testing after conditioning with THC (n = 8 – 11). In the graph B, JMV2959 was administered repeatedly together with THC during conditioning (n = 9 – 10). The results are presented as follows: saline + THC (open bar), JMV2959 1 mg/kg + THC (striped bar), JMV2959 3 mg/kg + THC (filled bar). The effect of conditioning with THC, thus the difference between pre- and post-conditioned measurements are expressed as * $p < 0.05$, *** $p < 0.001$. The effects of JMV2959 pretreatments in comparison to the saline group are expressed as # $p < 0.05$, ### $p < 0.001$. The results are presented as group means with 95% confidence intervals.

JMV2959 and ghrelin effects on WIN55,212-2 intravenous self-administration (IVSA) (main study)

We observed noticeable inter-individual differences in WIN55,212-2 IVSA activity among the rats during the whole study. For statistical evaluation, we have used the last three baselines before pretreatments as basal IVSA values. During the 3 days of saline pretreatment, the daily active lever-pressing ranged from 76 % to 194 % of baseline mean (see the Figure S2A). During the 3 days of JMV2959 pretreatment, the active lever-pressing ranged from 0% to 91% of baseline mean. With two exceptions, once at the 91 % on the first pretreatment session and once at the 58 % on the third pretreatment sessions, the JMV2959 active lever-pressing was below 37 %. Only in three sessions from all pretreatments the active lever-

pressing was completely abolished by the JMV2959 pretreatment (0 %). During the 3 sessions of ghrelin pretreatment, the active lever-pressing ranged from 131 % to 767 % of baseline mean. This is mainly because two rats were extremely interested in the active lever after ghrelin pretreatment and pressed above 541% of baseline mean (541 % - 767 %). Another two rats in the ghrelin group pressed above 300 % with maximum 345 % of baseline mean in at least two sessions, the active lever-pressing of the remaining rats reached maximum 261 % of baseline mean. The two rats with the highest numbers of active lever-pressing during all three pretreatments showed no apparent signs of behavioural disturbances, such as frozen postures, sedation etc., no back leaning on the lever, they were fully attracted to the active lever. These two rats did not differ from the rest of the rats considering the number of infusions (see Figure S2B). After ghrelin pretreatment, the number of infusions was ranging between 102% and 306% of baseline mean. Therefore, these rats achieved higher active lever-pressing during the time-out period. The JMV2959 pretreatment again reduced the number of infusions, thus increased the homogeneity of the values in the group.

Apparent differences in the individual reactivity of the rats to the pretreatments during the WIN55,212-2 seeking/relapse-like behaviour (on the 12th day of forced abstinence period) are illustrated in Figure S2C. The JMV2959 pretreatment reduced the non-rewarded cannabinoid-seeking/relapse-like active lever-pressing to values within 3 – 51 % of the baseline mean; active lever-pressing was never completely abolished on the relapse-test session. Within the ghrelin pretreated group, the unreinforced active lever-pressing was within 110 – 642 % of the baseline mean. Within the saline-group, the mean active lever-pressing ranged during the relapse-test session from 77% to 564 % of baseline mean, with average 189.6 % ± 52.7 of baseline mean, which indicates craving incubation in accordance with the literature [1].

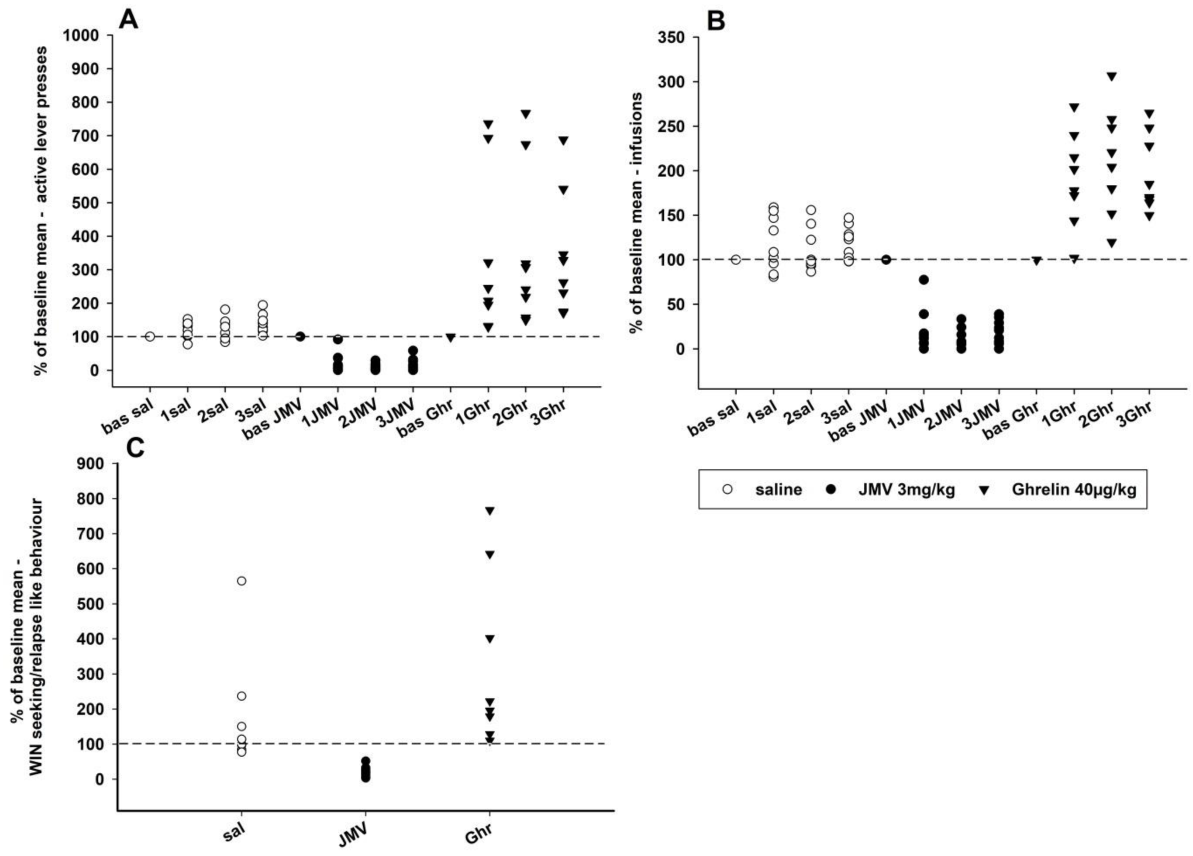

Fig. S2 Effects of JMV2959 and ghrelin on WIN55,212-2 intravenous self-administration (IVSA) in single rats in percentage of baseline mean (mean of the last three baselines before pretreatments, 5.-7. bas). Saline (1 ml/kg) or JMV2959 (3 mg/kg) or ghrelin (40 µg/kg) were administered intraperitoneally 20 min before the 120 min IVSA sessions. The active lever-pressing is presented in the graph **A**, the number of infusions in the graph **B** and the WIN55,212-2 –seeking/relapse-like non-reinforced active lever-pressing on the 12th day of forced abstinence during the relapse-test session in the graph **C**. The results are illustrated as follows: saline (open circle), JMV2959 (filled circle), ghrelin (filled triangle). The dotted line shows the baseline mean level (bas, 100%).

The body mass of rats within the IVSA study was daily measured and the changes during chosen periods are summarized in Table S1. The body mass changes were calculated within the main WIN55,212-2 IVSA experiment during the last week before pretreatments (bas 1 - 7), during the pretreatment days (JMV/sal/ghrelin 1 – 3), on the day of WIN55,212-2 -seeking/relapse-like behaviour testing and during all evaluated periods together (7 baselines + 3 JMV/sal/ghrelin + WIN55,212-2-seeking/relapse-like behavioural day = total 11 days). According to the two-way ANOVA RM/Bonferroni test, there were no significant differences

among the JMV2959, ghrelin and saline baseline groups as well as no influence of JMV2959/ghrelin/saline pretreatments. The only significant changes – weight gains were observed on the WIN55,212-2-seeking/relapse-like day in all three rat groups ($p < 0.001$), specifically 61 g within the JMV2959, 64 g within the saline and 59 g within the ghrelin group.

Changes of rat body mass within the main WIN55,212-2 IVSA study group means (g ± SEM)				
Group/interval	Baseline mean	Sal/JMV/ghrelin means	Relapse-test	Total mean
JMV2959 group	287.0 ± 3.0	292.2 ± 3.5	353.7 ± 2.8	294.5 ± 2.7
Saline group	288.1 ± 3.4	293.0 ± 6.5	357.0 ± 5.4	295.7 ± 3.7
Ghrelin group	287.4 ± 4.5	290.9 ± 6.0	349.7 ± 2.6	294.0 ± 4.3

Tab. S1. Changes of rat body mass within the IVSA study. The body mass changes are shown as group means ± SEM (JMV2959 group n = 10, Saline group n = 9, Ghrelin group n = 8) during the chosen periods of the experiment as follows: mean of the last 7 days before pretreatment (Baseline mean), mean of the 3 pretreatment days (Sal/JMV/ghrelin means), the day of the WIN55,212-2 -seeking/relapse-like behaviour test (Relapse-test), mean of 7 baseline days + 3 pretreatment days + day of WIN55,212-2 -seeking/relapse-behaviour testing (Total mean). Two-way ANOVA RM difference among the groups: F2,2 = 0.865, $p = 0.42$ (n.s.), effect of the time: F4,288 = 300.16; $p < 0.001$, (difference of WIN55,212-2 seeking/relapse-like behaviour in all groups - JMV2959, ghrelin and saline vs. other parameters), effect of the group x time: F4,288 = 0.255, $p = 0.91$ (n.s.).

JMV2959 and ghrelin effects on vehicle and WIN55,212-2 intravenous self-administration (IVSA) in additional study

A separate group of rats was used in an additional IVSA experiment for comparison of WIN55,212-2 IVSA and vehicle IVSA with the appropriate pretreatments. The remaining detailed changes within the number of infusions are illustrated in the Figure S3. The changes within the active lever-pressing were illustrated in the Figure 5. Comparison of the number of infusions using the two-way repeated measures ANOVA followed by Bonferroni test with factors: groups/IVSA type (WIN55.212-2/vehicle) and time (non pretreatment baseline/JMV2959/JMV2959+ghrelin/ghrelin) revealed significant differences among the groups (F1,6 = 3.84; $p < 0.05$), effect of the time (F3,18 = 20.12; $p < 0.001$) and the group x time effect (F3,18 = 9.86; $p < 0.001$). However, the pretreatments had no significant influence on the vehicle IVSA. Means of the three last baselines before pretreatments were

used in the statistical analysis. Within the cannabinoid IVSA, we observed significant reduction of number of infusions after JMV2959 pretreatment to 23.9% ± 5.0 of baseline mean ($p < 0.001$ in comparison to baseline). This JMV2959 effect was attenuated by ghrelin co-administration during the third pretreatment session to 58.13% ± 12.1(n.s. to baseline) and ghrelin pretreatment increased the number of infusions to 141.5 % ± 22.1 ($p < 0.05$ to baseline). When the changes were expressed in percentage of baseline mean (see the Figure S2B), two-way ANOVA RM/Bonferroni revealed again the significant pretreatment effects within the WIN55,212-2 IVSA group and no significant effects within the vehicle IVSA. Significant difference was found between WIN55,212-2 and vehicle IVSA in percentage of baseline means in number of infusions only in the co-administration (JMV2959 + ghrelin) session (p < 0.05) (effect of the time F2,12 = 28.19, p = 0.001; the group x time effect F2,12 = 11.4, p = 0.03; effect of the group was not significant, F1,6 = 2.81)

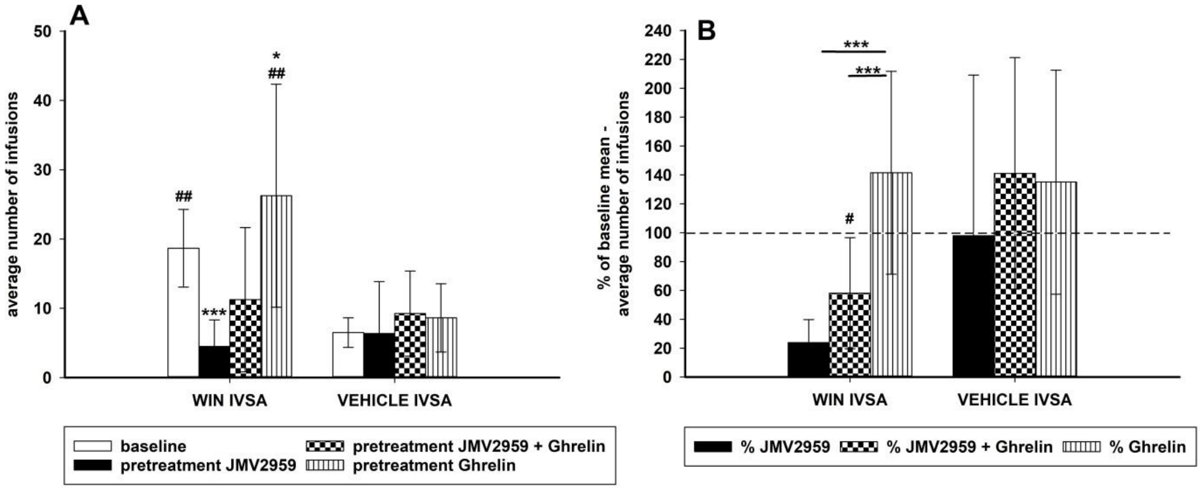

Figure S3. Additional IVSA experiment - effects of JMV2959 and ghrelin on number of infusions in the vehicle and WIN55,212-2 groups are illustrated in the graph A. The baseline number of infusions (mean of last three sessions before pretreatment) was influenced by pretreatment with JMV2959 (3 mg/kg) or JMV2959 + ghrelin or ghrelin (40 μg/kg) administered intraperitoneally 20 min before the 120 min sessions. The mean number of infusions are presented as follows: basal lever-pressing (open bar), JMV2959 (filled bar), JMV2959 + ghrelin (dotted bar), ghrelin (striped bar). Differences between WIN55,212-2 IVSA and vehicle IVSA are expressed as # $p < 0.05$, ## $p < 0.01$. Differences of pretreatments to baseline lever-pressing are expressed as * $p < 0.05$, *** $p < 0.001$. The effects of pretreatments illustrated in percentage of average baseline number of infusions (graph B) are presented as follows: percentage JMV2959 effect (filled bar), percentage JMV2959 + ghrelin effect (dotted bar), percentage ghrelin effect (striped bar). The statistical

differences between the percentage of WIN55,212-2 IVSA and vehicle IVSA are expressed as # $p < 0.05$. Differences between pretreatments are expressed as *** $p < 0.001$. Dotted line shows the baseline active lever-pressing (100%). The additional IVSA data went through logarithmic transformation before the statistical analysis, thus in the graphs are presented original data together with significances obtained from the transformed ANOVA results. The results are presented as group means with 95% confidence intervals ($n = 4$).

References:

1. Kirschmann, E. K.; Pollock, M. W.; Nagarajan, V.; Torregrossa, M. M., Effects of Adolescent Cannabinoid Self-Administration in Rats on Addiction-Related Behaviors and Working Memory. *Neuropsychopharmacology* **2017,** 42, (5), 989-1000.
